# QuickStats

**Published:** 2013-09-13

**Authors:** Steven M. Frenk, Yinong Chong

**Figure:**
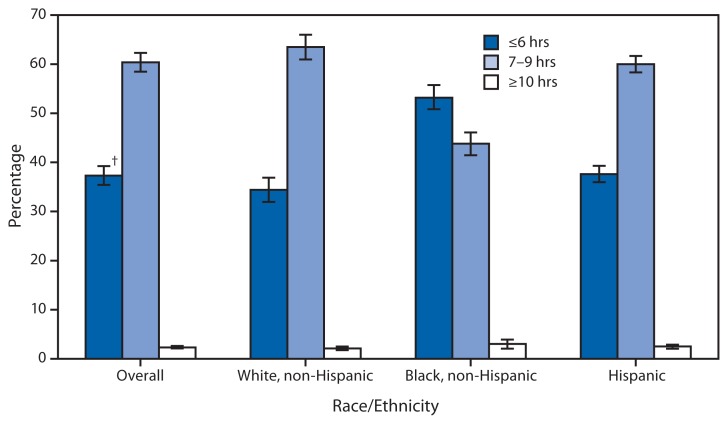
Sleep Duration* Among Adults Aged ≥20 Years, by Race/Ethnicity — National Health and Nutrition Examination Survey, United States, 2007–2010 * Data on sleep duration come from the question, “How much sleep do you usually get at night on weekdays or workdays?” All estimates are age-adjusted to the 2000 projected U.S. standard population using the age groups 20–39, 40–59, and ≥60 years. ^†^ 95% confidence interval.

During 2007–2010, 60.4% of U.S. adults aged ≥20 years slept 7–9 hours at night, 37.3% slept 6 hours or less, and 2.3% slept 10 hours or more. Non-Hispanic black adults were less likely to report sleeping 7–9 hours and more likely to report sleeping 6 hours or less than non-Hispanic white and Hispanic adults.

**Source:** CDC. National Health and Nutrition Examination Survey. Hyattsville, MD: US Department of Health and Human Services, CDC, National Center for Health Statistics; 2007–2010. Available at http://www.cdc.gov/nchs/nhanes.htm.

